# Coordination of Bactericidal and Iron Regulatory Functions of Hepcidin in Innate Antimicrobial Immunity in a Zebrafish Model

**DOI:** 10.1038/s41598-017-04069-x

**Published:** 2017-06-27

**Authors:** Xiao-feng Jiang, Zhi-fei Liu, Ai-fu Lin, Li-xin Xiang, Jian-zhong Shao

**Affiliations:** 1 College of Life Sciences, Zhejiang University, Hangzhou, 310058 People’s Republic of China; 2 Key Laboratory for Cell and Gene Engineering of Zhejiang Province, Hangzhou, 310058 People’s Republic of China; 3 Laboratory for Marine Biology and Biotechnology, Qingdao National Laboratory for Marine Science and Technology, Qingdao, People’s Republic of China

## Abstract

Hepcidin acts as both an antimicrobial peptide and a hormonal regulator of iron homeostasis; however, the biological significance of this dual-function in immune reactions remains elusive. In this study, we provide experimental evidence regarding the coordination of this dual-function in the innate antimicrobial immunity using a zebrafish model. The transcription of hepcidin gene was significantly upregulated in liver by *Aeromonas hydrophila* (*A.h*) DNA stimulation, which was accompanied by an increase of hepcidin protein and a decrease of iron concentration in serum. Thus, an enhanced bactericidal activity against *A.h* and *Escherichia coli* and inhibitory effects on *A.h* growth and OmpA expression were observed in *A.h* cells, the latter of which made the bacterium more susceptible to complement attack. The enhanced bacteriostatic activities in serum following the stimulation were dramatically impaired by neutralizing hepcidin or restoring iron to the samples. Immuno-protection assay showed that zebrafish administrated with *A.h* DNA or designed CpG-ODNs had a significantly enhanced defence against *A.h* and *Vibrio alginolyticus* infections, which was also eliminated by the neutralization of hepcidin. Results indicate that the induction of hepcidin leads to the decrease of iron in circulation, which eventually limits iron availability to invading microorganisms, thus contributing to host defence.

## Introduction

Hepcidin was originally identified as an antimicrobial peptide that contains 25 amino acids from human blood ultrafiltrate and is mainly produced by the liver and released into the serum^1, 2^. This peptide exhibits a strong bactericidal activity against various pathogenic bacteria, such as *Staphylococcus aureus*, *Aeromonas hydrophila*, and *Vibrio anguillarum* ^3, 4^. Hepcidin also plays a major role in the control of iron homeostasis in the body and serves as a key regulator of iron balance and recycling in humans and mice^5, 6^. Hepcidin regulates iron homeostasis by inhibiting cellular iron efflux through an interaction with ferroportin (Fpn) and inducing the internalization of Fpn, which is the only known transmembrane iron exporter expressed in enterocytes, macrophages, and hepatocytes^7^. Therefore, iron absorption in the intestines and the serum iron concentration are decreased^8^. These observations indicate that hepcidin is a bactericidal peptide and an iron regulatory hormone that plays a dual function in the host innate immunity and iron metabolism. Thus, hepcidin provides a connection between anti-bacterial infections and host iron metabolism^9^. Evolutionarily, hepcidin has likely evolved from an antimicrobial peptide to an iron regulatory hormone in ancient vertebrates because its structural and functional characterizations and transcriptional regulatory mechanisms have remained highly conserved among different species throughout vertebrate evolution^10^. However, to date, the biological significance of the evolution of hepcidin that created a direct link between innate immunity and iron metabolism has not been fully elucidated.

Physiologically, iron is an essential element for almost all living organisms, including bacteria, humans, and other mammals^11^. In bacterial infections, pathogenic microorganisms capture iron from the host transferrin or lactoferrin via its iron-carrying proteins, which have a higher iron affinity than that of the other two host proteins^10, 12^. Thus, the depredation of iron in the host has become an important strategy for bacterial growth and survival^13, 14^. In host defence against bacterial invasions, the complement system, which includes the classical, lectin, and alternative pathways, plays important roles in innate and adaptive immunities^15^. Upon a bacterial infection, the pathogen-associated molecular patterns (PAMPs) on the bacterial surface activate the lectin and alternative pathways, while the antibodies adsorbed on the bacterial cell walls activate the classical pathway^16^. The activation of the complement pathways leads to the formation of a membrane attack complex, which is critical for the lysis of invading bacteria^17^. Bacteria, in turn, escape from the complement attack by expressing an outer membrane protein A (OmpA) on their cellular surfaces^18^. OmpA can strongly bind to the host C4bp and Factor I to form a complex that degrades C3b and C4b and, thus, inactivates the complement pathways^19, 20^. OmpA expression in bacteria is closely correlated with iron content^21, 22^. These findings suggest that iron is not only essential to bacterial growth, but also has a regulatory role in the defence of bacteria against host immunity. Therefore, the occurrence of the dual function of hepcidin, including its bactericidal activity and iron regulatory capacity, may be a remarkable biological event that improves the antimicrobial activity of this peptide by creating a new iron regulatory pathway. In this potential new pathway, alterations in iron metabolism likely function in the host defence by limiting the availability of iron to the invading microorganisms. In the present study, experimental lines were provided to support this hypothesis using a zebrafish model and to enhance our understanding of the biological characteristics of hepcidin and the evolutionary history of the antibacterial innate immunity as a whole.

## Results

### Increased liver-derived hepcidin and decreased serum iron following stimulation by *A. hydrophila* DNA

**Figure 1:**
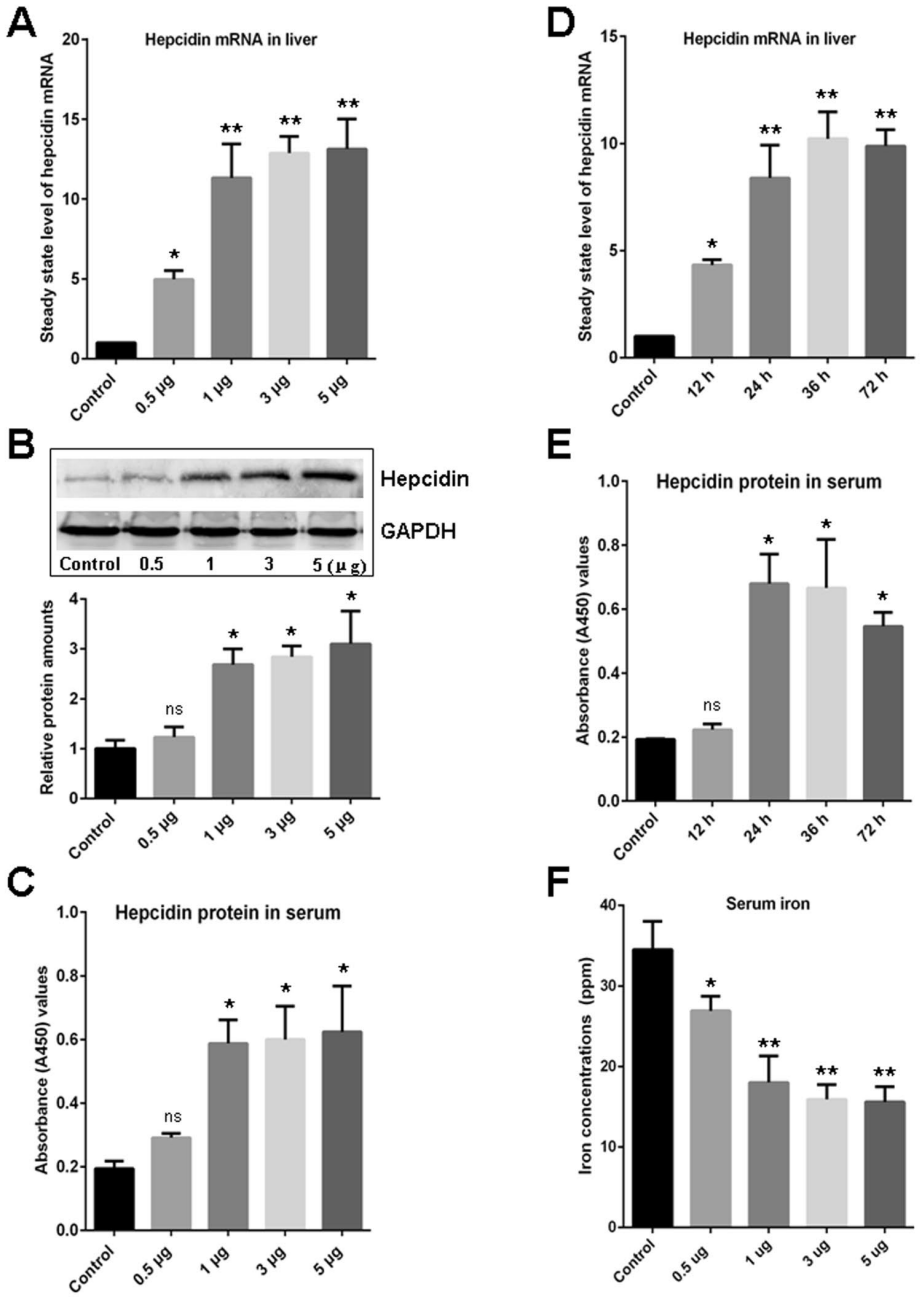
Evaluation of the induced expression of hepcidin and reduced serum iron concentration upon *A. hydrophila* DNA stimulation. (**A**) Detection of the steady state level of hepcidin mRNA in liver upon stimulation with various concentrations of *A. hydrophila* DNA (0.5, 1, 3, and 5 μg per fish) by Q-PCR. (**B** and **C**) Detection of the hepcidin protein level in serum upon stimulation with various concentrations of *A. hydrophila* DNA (0.5, 1, 3, and 5 μg per fish) by Western blot (**B**) and ELISA (**C**), and the gray value of the relative signal intensity (hepcidin/GAPDH) in Western blot analysis was calculated using ImageJ program and depicted in a bar graph. (**D**) Time-dependent upregulation of the steady state level of hepcidin mRNA in liver upon stimulation with *A. hydrophila* DNA (1 μg per fish) as determined by Q-PCR. (**E**) Time-dependent upregulation of the hepcidin protein in serum upon stimulation with *A. hydrophila* DNA (1 μg per fish) as determined by ELISA. (**F**) Reduced concentrations of serum iron stimulated by *A. hydrophila* DNA (0.5, 1, 3, and 5 μg per fish) were detected by ICP-MS. The control groups in each experiment (indicated as Control) received mock PBS. The steady state level of hepcidin mRNA in liver was calculated by 2^−ΔΔCT^ method normalized to β-actin. All data are representative of at least three independent experiments. A significant difference was detected between each experimental and control group. *p < 0.05, **p < 0.01. ns for not significant. The full-length blots are presented in Supplementary Fig. S4.

Bacterial genomic DNA that includes the stimulating CpG-motifs is an important PAMP that can elicit innate immune responses via the TLR9 signaling pathway. In our study, bacterial DNA from *Aeromonas hydrophila* (*A. hydrophila*) was used as an infectious stimulus to evaluate the association between the induced upregulation of hepcidin and the amount of peripheral serum iron in the circulation following this infectious stimulation. As expected, the steady state level of hepcidin mRNA in the liver (12-fold induction as determined by Q-PCR) and the amount of hepcidin protein in the serum (3-fold induction as determined by Western blot and ELISA) of the experimental fish that were administered with various concentrations of *A. hydrophila* DNA were remarkably (p < 0.01) higher than that in the unstimulated control fish (Fig. 1A,B, and C). The hepcidin was induced in a dose-dependent manner and maintained at a higher level from 24 h to 36 h after the stimulation (Fig. 1D and E). During this period, the amount of iron in the serum samples that were collected from the same stimulated fish was significantly (p < 0.01) decreased from 34.5 ± 3.5 ppm to 15.6 ± 1.9 ppm compared with that in the control group, as detected by ICP-MS. The serum iron concentration in response to the *A. hydrophila* DNA stimulation also decreased in a dose-dependent manner (Fig. 1F). A cross-correlation (CORREL) analysis revealed that the increased amount of hepcidin protein in the serum was inversely correlated (−0.99629) with the declined serum iron concentration following the *A. hydrophila* DNA stimulation.

### Enhanced bacteriostatic ability in the serum from the *A. hydrophila* DNA-stimulated fish

**Figure 2:**
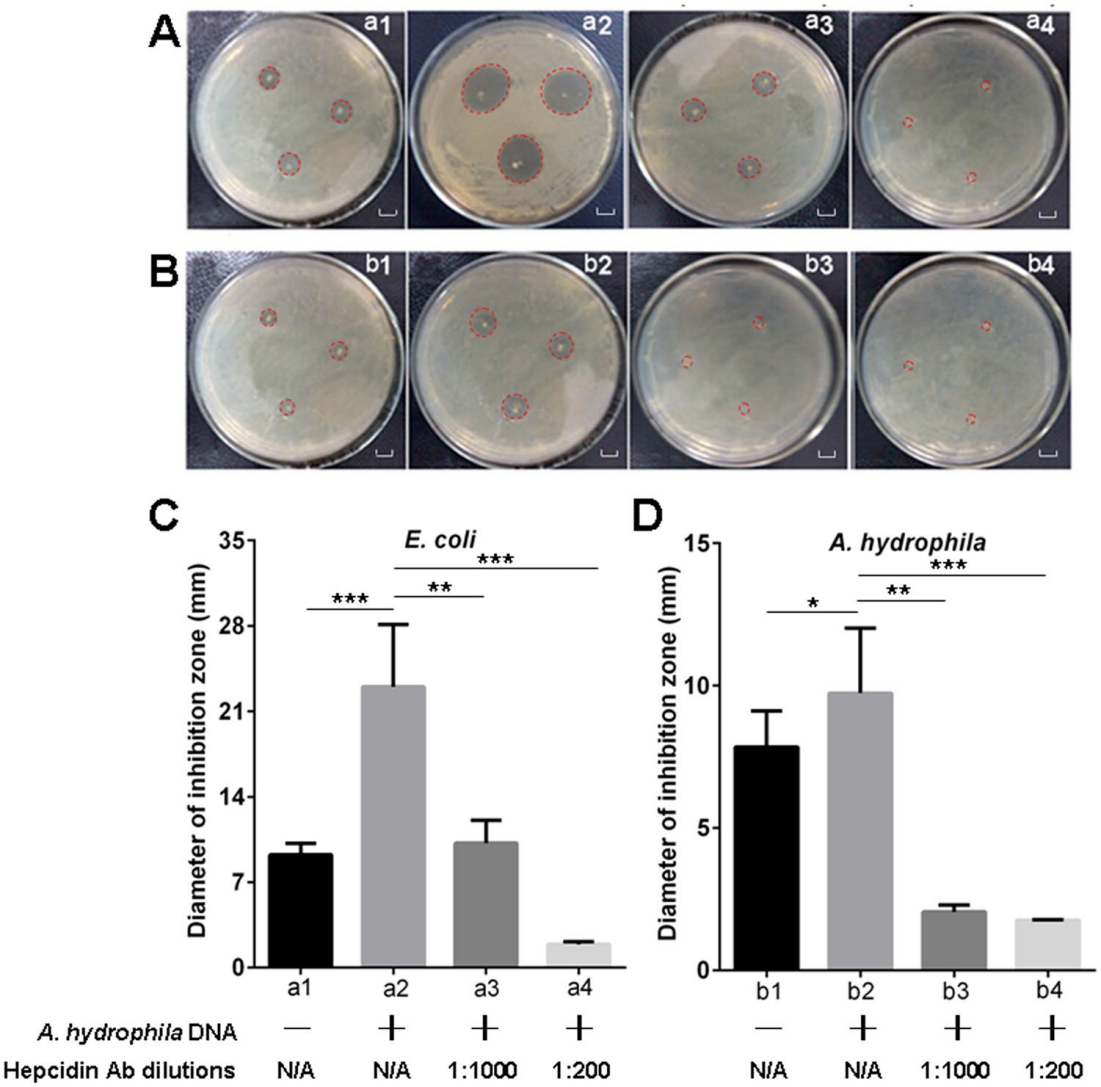
Evaluation of the bactericidal activity of hepcidin in various serum samples against test strains by inhibition zone assay. (**A** and **B**) Detection of the inhibition zones for *E. coli* (**A**) and *A. hydrophila* (**B**) strains by unstimulated serum (a1 and b1), *A. hydrophila* DNA (1 μg per fish) stimulated serum (a2 and b2), *A. hydrophila* DNA stimulated serum neutralized with diluted (1:1,000) anti-hepcidin Ab (a3 and b3), and *A. hydrophila* DNA stimulated serum neutralized with diluted (1:200) anti-hepcidin Ab (a4 and b4), scale bars = 1 cm. (**C** and **D**) Measurement of the corresponding diameters of the inhibition zones (mm) from the inhibition zone assays by using serum samples with various treatments against *E. coli* (**C**) and *A. hydrophila* (**D**) strains. The labels a1 to a4 and b1 to b4 under the horizontal axis of C and D are in accordance with **A** and **B**. The annotations “*A*. *hydrophila* DNA+/−” and “Hepcidin Ab dilutions” under the labels represent that the *E*. *coli* (a1 to a4) and *A*. *hydrophila* (b1 to b4) were treated with the serum derived from *A*. *hydrophila* DNA stimulated (+) or unstimulated (−) fish, which were in combination with neutralizing the serum with anti-hepcidin Ab at different dilutions (1:1000 and 1:200) or without treatment with the anti-hepcidin Ab (N/A, not applicable). Data are representative of at least three independent experiments. *p < 0.05, **p < 0.01, ***p < 0.001.

**Figure 3:**
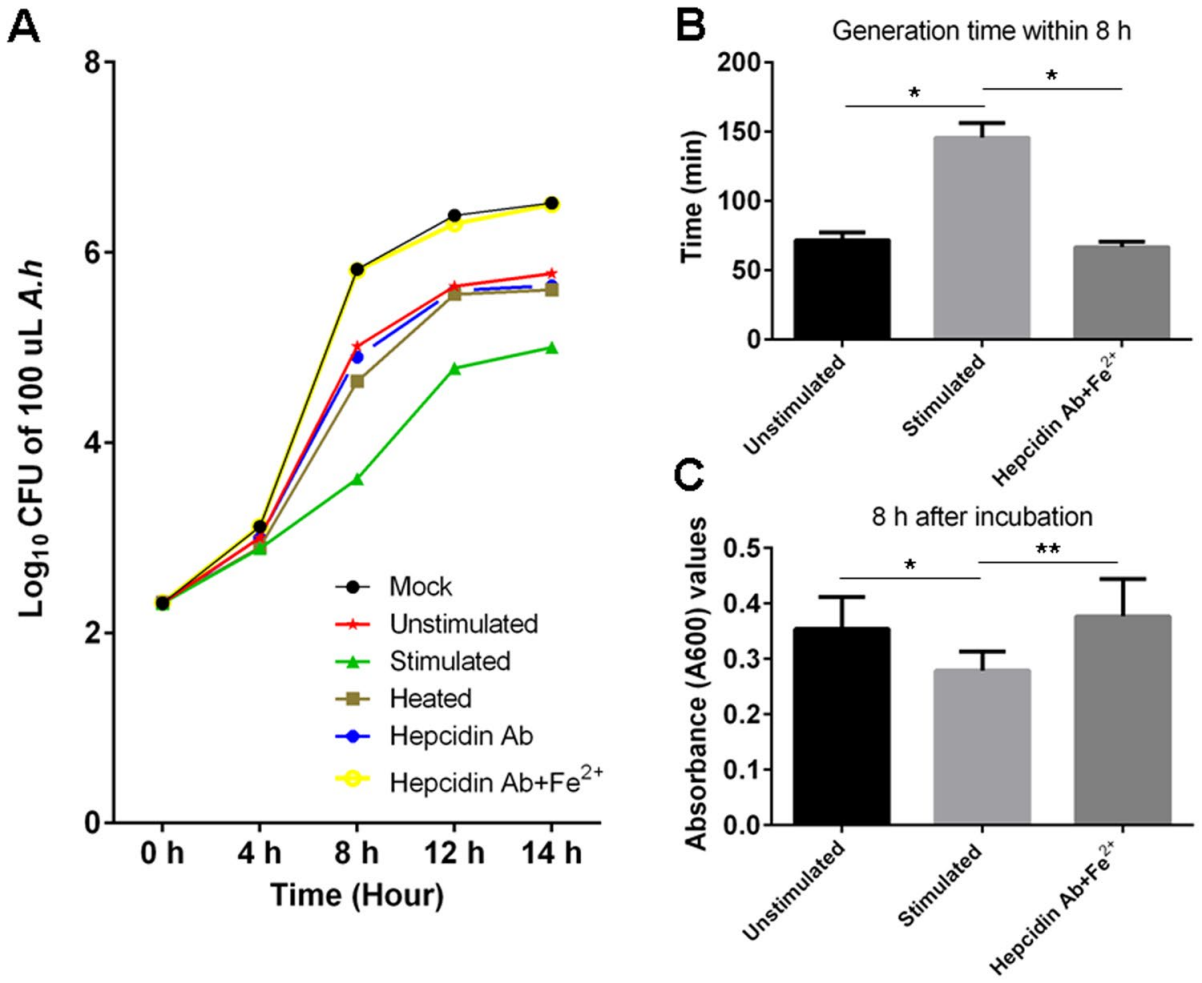
Inhibitory effect of serum derived from *A. hydrophila* DNA stimulated zebrafish on the growth of *A. hydrophila*. (**A**) Growth curve of *A. hydrophila* (*A.h*) in M9 medium cultured in the presence of unstimulated serum (Unstimulated), *A. hydrophila* DNA (1 μg per fish) stimulated serum (Stimulated), heat-treated stimulated serum (Heated), stimulated serum neutralized with anti-hepcidin Ab (Hepcidin Ab), stimulated serum neutralized with anti-hepcidin Ab plus 10 μM Fe^2+^ supplemented ones (Hepcidin Ab + Fe^2+^), and *A. hydrophila* cultured alone in M9 (Mock). (**B**) Detection of the bacterial generation time of *A. hydrophila* cultured in the presence of different serum samples (unstimulated, stimulated, and Hepcidin Ab + Fe^2+^) in M9 medium for 8 h. (**C**) Detection of the absorbance (A_600_) values of *A. hydrophila* cultured in the presence of different serum samples (unstimulated, stimulated, and Hepcidin Ab + Fe^2+^) in M9 medium at 8 h time point. All data are representative of at least three independent experiments. *p < 0.05, **p < 0.01.

The serum samples with an increased amount of hepcidin protein and a decreased iron content from the *A. hydrophila* DNA-stimulated fish (defined as stimulated serum) were used in our case study to evaluate whether the upregulated serum hepcidin protein and decreased iron content in the peripheral circulation following the infectious stimulation act as a dichotomous strategy in antibacterial innate immunity. The enhanced bactericidal activity, which was derived from the increased hepcidin content and the improved inhibitory effect on bacterial growth, which resulted from the decreased iron, were detected. The bactericidal activity of the stimulated serum against *Escherichia coli* (*E. coli*) and *A. hydrophila* was significantly higher (p < 0.01) than that of the normal serum from the unstimulated fish, as determined by an increase in the diameter of the inhibitory zones from 9.7 ± 0.9 mm to 22.1 ± 5.8 mm for *E*. *coli* and from 3.7 ± 0.7 mm to 9.0 ± 1.2 mm for *A. hydrophila* (agar diffusion method; Fig. 2). In contrast, the enhanced bactericidal ability of the stimulated-serum was significantly impaired (p < 0.01) because of the increased concentrations of anti-hepcidin Ab, as revealed by the shorter diameter of the inhibitory zones in the antibody-neutralized groups compared with that in the untreated control group (Fig. 2A and B). This result suggested that the upregulation of the serum hepcidin protein level may greatly contribute to the enhanced bactericidal activity in the serum from the stimulated fish. In the iron-dependent growth inhibition assay, the stimulated serum was diluted in an iron-free M9 medium, incubated at 56 °C for 1 h, and treated with anti-hepcidin Ab. The serum was subsequently subjected to immunoprecipitation (IP) and ultrafiltration treatments to remove the potential activities of hepcidin and the complement components. The results showed that the growth of *A. hydrophila* in the M9 medium with the pre-treated stimulated serum was significantly inhibited (p < 0.01) by almost 2-fold between 8 h to 14 h compared with that in the medium with the normal control serum from the unstimulated fish as determined by the decreased colony-forming units (CFU) on the LB agar medium plates (Fig. 3A). The decreased bacterial growth could be significantly restored (p < 0.05) by supplementing the medium with iron (10 μM Fe^2+^) into the medium (Fig. 3B and C). These results suggested that the decreased iron content in the stimulated serum likely contributes to the inhibition of bacterial proliferation.

### Impaired bacterial resistance to a complement attack in the *A*. *hydrophila* DNA-stimulated serum

**Figure 4:**
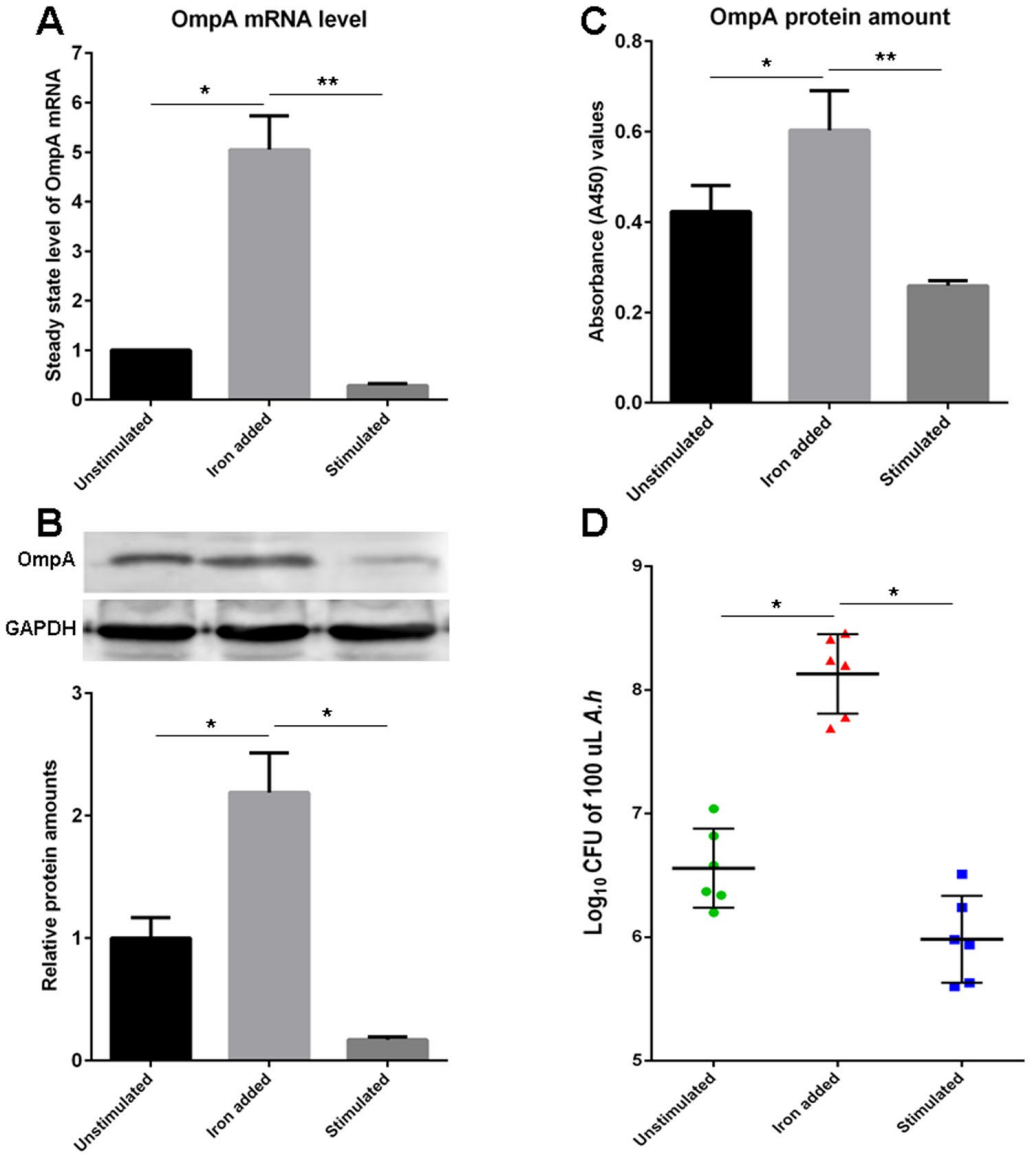
Evaluation of OmpA expression on *A. hydrophila* cells upon iron-deficient serum stimulation and bacterial susceptibility to complement attack. (**A**, **B**, and **C**) The steady state level of OmpA mRNA or the amount of OmpA protein in *A. hydrophila* cells cultured in the presence of unstimulated serum (Unstimulated), *A. hydrophila* DNA (1 μg per fish) stimulated serum plus 10 μM Fe^2+^ supplemented ones (Iron added), and *A. hydrophila* DNA stimulated serum (Stimulated) was detected by Q-PCR (**A**), Western blot (**B**) and ELISA (**C**). The gray value of the relative signal intensity (OmpA/GAPDH) in Western blot analysis (**B**) was calculated using ImageJ program and depicted in a bar graph. All data are representative of at least three independent experiments. (**D**) Evaluation of the log_10_ CFU of 100 μL *A. hydrophila* under the attack of guinea pig serum (diluted to 5% as the complement source). *A. hydrophila* cultured in the presence of differently treated serum samples followed by co-incubation with guinea pig serum. The green round spots (Unstimulated) showed Log_10_ CFU of 100 μL *A. hydrophila* incubated with normal serum (n = 6), the red triangles (Iron added) showed Log_10_ CFU of 100 μL *A. hydrophila* incubated with *A. hydrophila* DNA stimulated serum added with 10 μM iron (n = 6), and the blue squares (Stimulated) showed Log_10_ CFU of 100 μL *A. hydrophila* incubated with *A. hydrophila* DNA stimulated serum (n = 6). The bars in the middle of each group mean average value. The steady state level of OmpA mRNA in *A*. *hydrophila* cells was calculated by 2^−ΔΔCT^ method normalized to 16 s rRNA. *p < 0.05, **p < 0.01. The full-length blots are presented in Supplementary Fig. S5.

Considering that OmpA helps bacteria evade the recognition and attack by the host complement systems, whose expression is highly regulated by iron, we then explored whether the expression of OmpA was inhibited in the iron-deficient serum from the *A. hydrophila* DNA-stimulated fish. Our results indicated that the susceptibility of the bacteria to a complement attack was increased. The steady state level of OmpA mRNA and the amount of OmpA protein were significantly lower (p < 0.05) in the *A*. *hydrophila* cells that was incubated with iron-deficient serum than those in the normal control serum-incubated group, as demonstrated by Q-PCR, Western blot and ELISA, respectively (Fig. 4A,B and C). The decreased bacterial OmpA mRNA levels and protein amounts in the iron-deficient serum-incubated groups can be significantly restored (p < 0.01) by adding iron (10 μM Fe^2+^) to the serum samples. Correspondingly, the susceptibility of *A. hydrophila* to an attack by a guinea pig serum complement in the iron-deficient serum-treated groups was higher (p < 0.05) than that in the normal control serum-incubated groups as determined by the significant decline in the CFU of *A. hydrophila* in the experimental groups (Fig. 4D). Similarly, the increased susceptibility of *A. hydrophila* to a complement attack in the iron-deficient serum-treated groups can also be significantly restored (p < 0.05) by adding iron (10 μM Fe^2+^) to the serum as shown by the decreased CFU in the bacterial growth assay. These results suggested that the iron deficiency reduced the OmpA expression on the bacterial outer membranes. Thus, the resistance of the bacteria to the complement attack was reduced.

### *In vivo* evaluation of the hepcidin-mediated defence against bacterial infections

**Figure 5:**
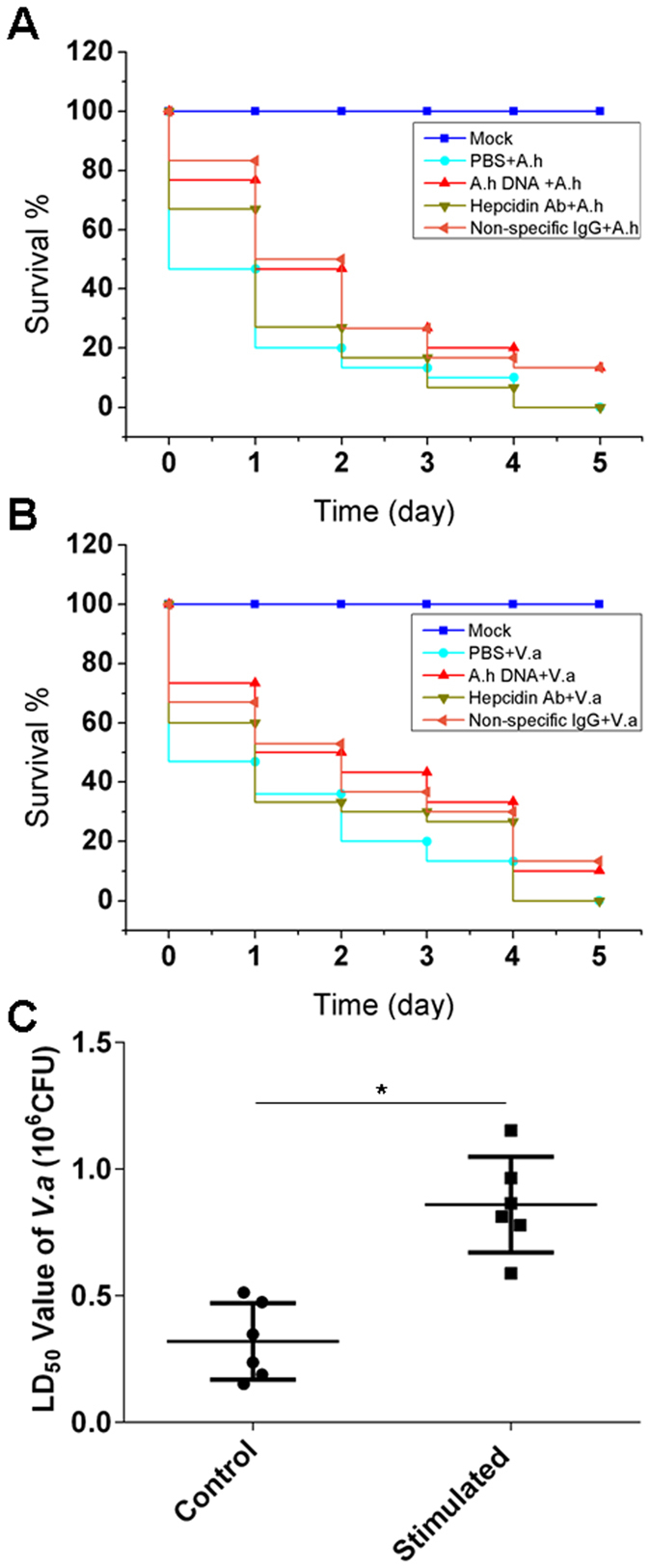
*In vivo* immunoprotection assay of hepcidin-mediated antibacterial activity. (**A**) The survival rate of zebrafish upon *A. hydrophila* (*A.h*) infection (2.1 × 10^5^ CFU per fish). The fish received various treatments before infection, including stimulation with *A. hydrophila* DNA (1.5 μg per fish, indicated as *A.h* DNA + *A.h*), administration of anti-hepcidin Ab for hepcidin neutralization (indicated as Hepcidin Ab + *A.h*), in which the non-specific rabbit IgG was administrated into the *A. hydrophila* DNA stimulated fish as a negative control (indicated as Non-specific IgG + *A.h*), and administration of mock PBS into the non-stimulated control fish (indicated as PBS + *A.h*). (**B**) The survival rate of zebrafish upon *V. alginolyticus* (*V.a*) infection (2.9 × 10^5^ CFU per fish). The fish received various treatments before infection similar to that as described in Fig. 5A. The Mock fish (indicated as Mock in Fig. 5A and B) received only *A*. *hydrophila* DNA without infections. (**C**) Immunoprotective effect of hepcidin-mediated antibacterial immunity against *V. alginolyticus* infection determined by the alteration of lethal dose 50 (LD_50_) of *V. alginolyticus* that kills 50% of the test fish within a set amount of time (24 h). The control fish (indicated as Control) received only PBS before infections. The stimulated fish (indicated as Stimulated) received *A*. *hydrophila* DNA (1.5 μg per fish). At least 30 fish were used in each experimental group, and all data are representative of at least three independent experiments. *p < 0.05.

An *in vivo* immunoprotection assay was performed to examine the hepcidin-mediated defence against the bacterial infection that was elicited by the *A. hydrophila* DNA stimulation. The survival of zebrafish in each group was detected each day during the 5-day experimental period. The results showed that the zebrafish that did not receive the *A. hydrophila* DNA stimulation (control group) significantly exhibited to be lethal to both *A. hydrophila* and *Vibrio alginolyticus* (*V. alginolyticus*) infections, as determined by the dramatic decline in survival in each day post-infection (46.7% ± 6.7%, 20.0% ± 3.3%, 13.3% ± 6.7%, 10.0% ± 6.7% and 0% for *A*. *hydrophila*; and 46.7% ± 10.0%, 33.3% ± 6.7%, 20.0% ± 3.3%, 0% for *V*. *alginolyticus*) (Fig. 5A and B). The mortality of the control fish after the infection was significantly higher (p < 0.05, Breslow test) than that of the stimulated fish within 5 days, and a rapid peak in mortality was observed within 2 days. Approximately 20% (20.0% ± 3.3%) of the zebrafish survived 2 days after *A. hydrophila* infection and less than 35% (33.3% ± 6.7%) of the zebrafish survived 2 days after *V. alginolyticus* infection. All of the control fish died within 5 days of the infection, and the accumulated survival rate within the first 4 days of infection was 10% ± 3.3%. By contrast, *A. hydrophila* DNA-stimulated zebrafish with a higher upregulation of hepcidin and a lower serum iron concentration exhibited a relatively smooth disease progression with a significant higher survival (76.7% ± 13.3%, 46.7% ± 3.3%, 26.7% ± 3.3%, 20.0% ± 6.7% and 13.3% ± 3.3% for *A*. *hydrophila* infection; and 73.3% ± 3.3%, 50.0% ± 6.7%, 43.3% ± 6.7%, 36.7% ± 3.3% and 10.0% ± 3.3% for *V*. *alginolyticus* infection) than that of the control fish at each of the examined time points (Fig. 5A and B). The survival rate in control fish was increased by more than 135% seen at 2 days of the peak mortality time, and the accumulated survival rate within the first 4 days of infection was increased by almost 150% relative to that of the control group. Approximately 10% of *A. hydrophila* DNA-stimulated fish remained alive 5 days after infection, but all of the control fish died in this period. However, administration of anti-hepcidin Ab remarkably impaired (p < 0.05, Breslow test) the immunoprotection of fish to bacterial infection stimulated by *A. hydrophila* DNA, as shown by more than 50% and 20% decreases in survival rate at 2 days after *A. hydrophila* and *V. alginolyticus* infections, among which the survival rate in *V. alginolyticus*-infected group was even lower than that of the control group. In contrast, there was no significant inhibitory effect on the immunoprotection of *A. hydrophila* DNA stimulated fish that received non-specific IgG Ab (normal rabbit IgG polyclonal Ab from naive rabbit serum that was used as a negative control). These observations indicated that the neutralization of hepcidin reduced the antibacterial activity of zebrafish, suggesting that hepcidin-mediated defence mechanisms play major roles in host innate antibacterial immunity. Similar results were also seen in another immunoprotection assay determined by the alterations of lethal dose 50 (LD50), in which *A. hydrophila* DNA-stimulated zebrafish tolerated more dosages of *V*. *alginolyticu*s challenge than the control group (Fig. 5C). These results suggested that hepcidin augments the host defence against a bacterial infection following bacterial DNA stimulation.

### A CpG-ODN-stimulated immunoprotection strategy against bacterial infection

**Figure 6:**
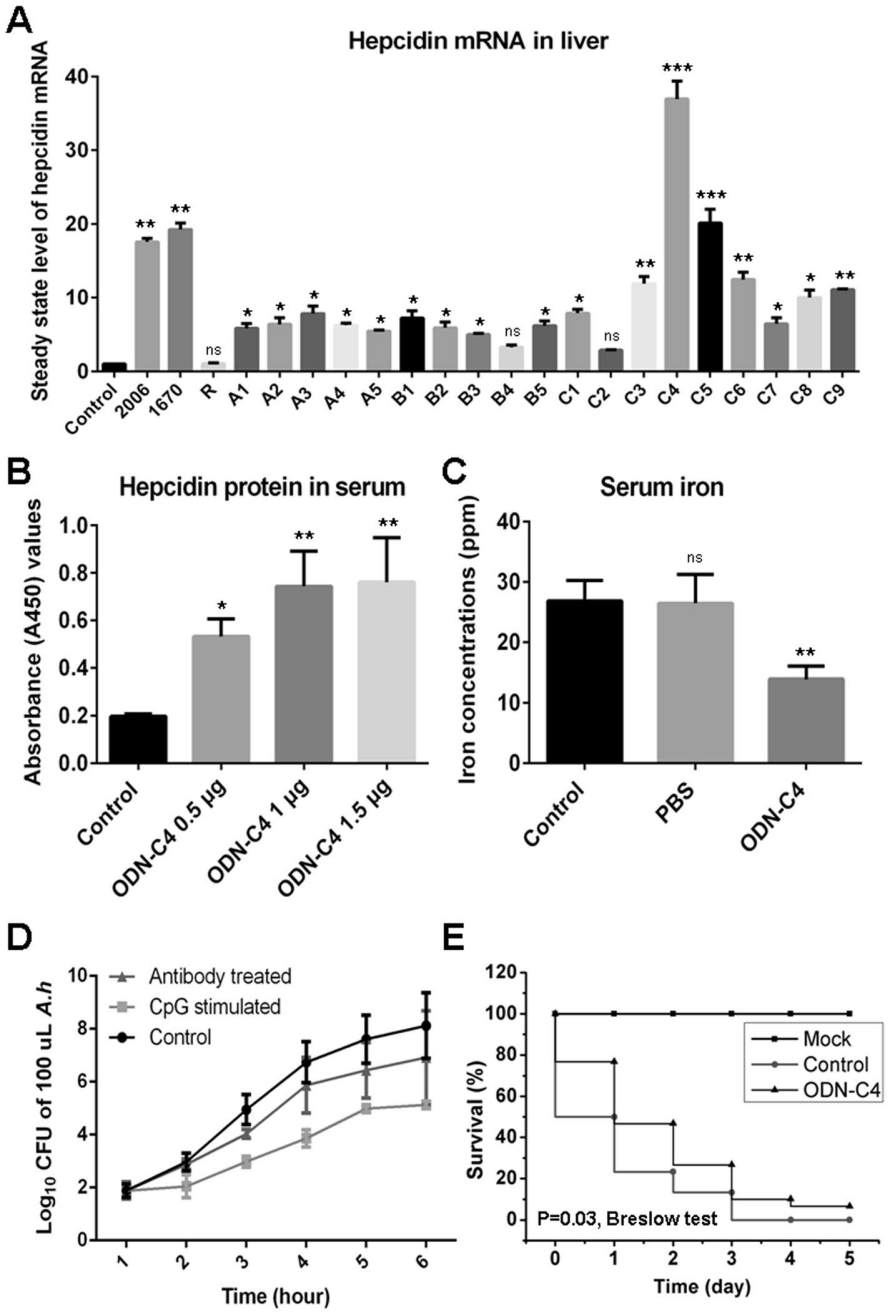
Screen of CpG-ODNs for hepcidin induction and its immunoprotection to bacterial infection. (**A**) The steady state level of hepcidin mRNA in liver upon various CpG-ODNs stimulation as determined by Q-PCR. The control fish (indicated as Control) received mock PBS. The sequences of the three known CpG-ODNs (2006, 1670 and R) and the candidate CpG-ODNs with Class A (A1–A5), Class B (B1–B5) and Class C (C1–C9) stimulatory motifs was shown in Supplementary Table S1. (**B**) Dose-dependent effect of ODN-C4 on the induction of hepcidin protein in serum as determined by ELISA, A_450_ represents absorbance (A_450_) value. The control fish (indicated as Control) received mock PBS. (**C**) Detection of iron concentration in serum of fish receiving ODN-C4 stimulation as determined by ICP-MS. Serum sample derived from the zebrafish without any treatment was the control group (indicated as Control), and serum sample derived from the mock PBS-injected zebrafish was the PBS group (indicated as PBS). (**D**) Examination of *A. hydrophila* (*A.h*) growth in M9 medium cultured in the presence of unstimulated serum (indicated as Control), 1.5 μg ODN-C4 stimulated serum (indicated as CpG stimulated) and stimulated serum neutralized with diluted (1:1,000) anti-hepcidin Ab (indicated as Antibody treated). (**E**) Survival rate of ODN-C4 stimulated fish within 5 days after *A*. *hydrophila* infection. The mock group received only ODN-C4 without bacterial challenge (indicated as Mock) and the control group received sterile PBS 24 h before the bacterial challenge (indicated as Control). All data are representative of at least three independent experiments. A significant difference was detected between each experimental and control group. *p < 0.05, **p < 0.01, ***p < 0.001. ns for not significant.

The above-mentioned results showed that the bacterial genomic DNA is a strong stimulant for the induction of hepcidin in zebrafish. For therapeutic application purposes, we attempted to develop a substitute for the bacterial genomic DNA with greater stimulatory efficacy and biological safety using CpG-deoxynucleotides (CpG-ODNs), which are typical PAMP stimulants of bacterial/viral genomic DNA that are capable of inducing host innate immune responses via toll-like receptor 9 (TLR9). In total, 19 phosphorothioate-modified CpG-ODN candidates with stimulatory CpG motifs (Supplementary Table S1) were designed and synthesized for the functional examination. The two known stimulatory CpG-ODNs (ODN-1670 and ODN-2006) and a CpG-R with an inverted CpG were used as the positive and negative controls, respectively^23, 24^. After several rounds of screening by detecting the induced steady state level of hepcidin mRNA among the 19 CpG-ODNs, the ODN-C4 fragment was confirmed to have the most effective inducibility. This fragment induces the upregulation of hepcidin by 47 folds in the liver at mRNA level and 5 folds in the serum at protein level compared with that in the unstimulated control fish, which was significantly higher (p < 0.01) than that induced by the other candidates. Correspondingly, the amount of the iron in the serum samples that were collected from the ODN-C4-stimulated fish was significantly decreased (p < 0.01; from 33.5 ± 2.7 ppm to 12.6 ± 1.4 ppm) compared with that in the unstimulated and mock PBS-treated control groups (Fig. 6A–C). Meanwhile, we also found that the growth of *A. hydrophila* in the serum from the ODN-C4-stimulated fish was significantly inhibited (p < 0.01) compared with that in control serum, and this inhibition could be dramatically eliminated (p < 0.01) by incubating the serum sample with anti-hepcidin Ab (Fig. 6D). Consistent with the above observations, the immunoprotection assay showed that the accumulated survival rate of the ODN-C4 injected group was significantly (*P* < 0.05, Breslow test) increased by 80% compared with that of the control group (fish that only received mock PBS before infection) in the first 3 days after the infection with *A*. *hydrophila* (Fig. 6E), and the zebrafish that only received ODN-C4 without the infection (Mock) did not show an effect on mortality. The results demonstrated that CpG-ODNs can be strong stimulants for hepcidin induction; thus, they are expected to become a new antibacterial agent based on the enhanced hepcidin-mediated innate immunity.

## Discussion

Hepcidin is a cationic amphipathic peptide that is synthesized mainly by hepatocytes, released into the plasma, and excreted in the urine. It acts as both a bactericidal peptide and a homeostatic regulator of intestinal iron absorption, iron recycling by macrophages, and iron mobilization from hepatic stores^25^. Iron, however, is a principal element required for bacterial growth and escape from a complement attack by induced OmpA expression^20^. These observations suggested a cooperative effect between the antibacterial and iron regulatory activities of hepcidin in the innate immune defence against bacterial invasion. In our study, using a zebrafish model, we provided experimental lines supporting this hypothesis. First, the steady state level of hepcidin mRNA in liver and the amount of hepcidin protein in serum were significantly upregulated following stimulation by *A. hydrophila* DNA, and the induced upregulation of hepcidin was accompanied by a decrease in the iron content in the serum. The increase of serum hepcidin protein was significantly and inversely correlated with the decrease of serum iron following the stimulation. Second, the serum with the increased hepcidin and decreased iron yielded an enhanced bacteriostatic ability as shown by its enhanced bactericidal activity and bacterial growth inhibition. This enhanced bacteriostatic ability could be remarkably impaired by the neutralization of hepcidin through the incubation of the serum with anti-hepcidin Ab. This finding suggested that hepcidin plays an important role in the innate antibacterial immunity. Third, the expression of OmpA protein on the bacterial outer membrane was inhibited by incubating the cells with the iron-deficient serum. The bacteria with the decreased OmpA protein were more susceptible to the attack by the complement system. Meanwhile, we also found that the decrease of OmpA protein would lead to a significant weakening in pathogenicity of *A*. *hydrophila* (Supplementary Fig. S3). The immunoprotection assay demonstrated that the zebrafish that were stimulated by the *A. hydrophila* DNA or CpG-ODNs stimulation had a significantly enhanced defence against bacterial infection, and this enhancement could be remarkably eliminated by the removal of hepcidin through the administration of the anti-hepcidin Ab. These results elucidated the *in vivo* mechanism of the hepcidin-mediated innate antimicrobial immunity, and further indicated that the induction of hepcidin in the liver caused a decrease in the iron levels in circulation and consequently reduced the extracellular iron concentrations. Thus, iron availability to invading microorganisms became limited and the host defence was improved.

**Figure 7:**
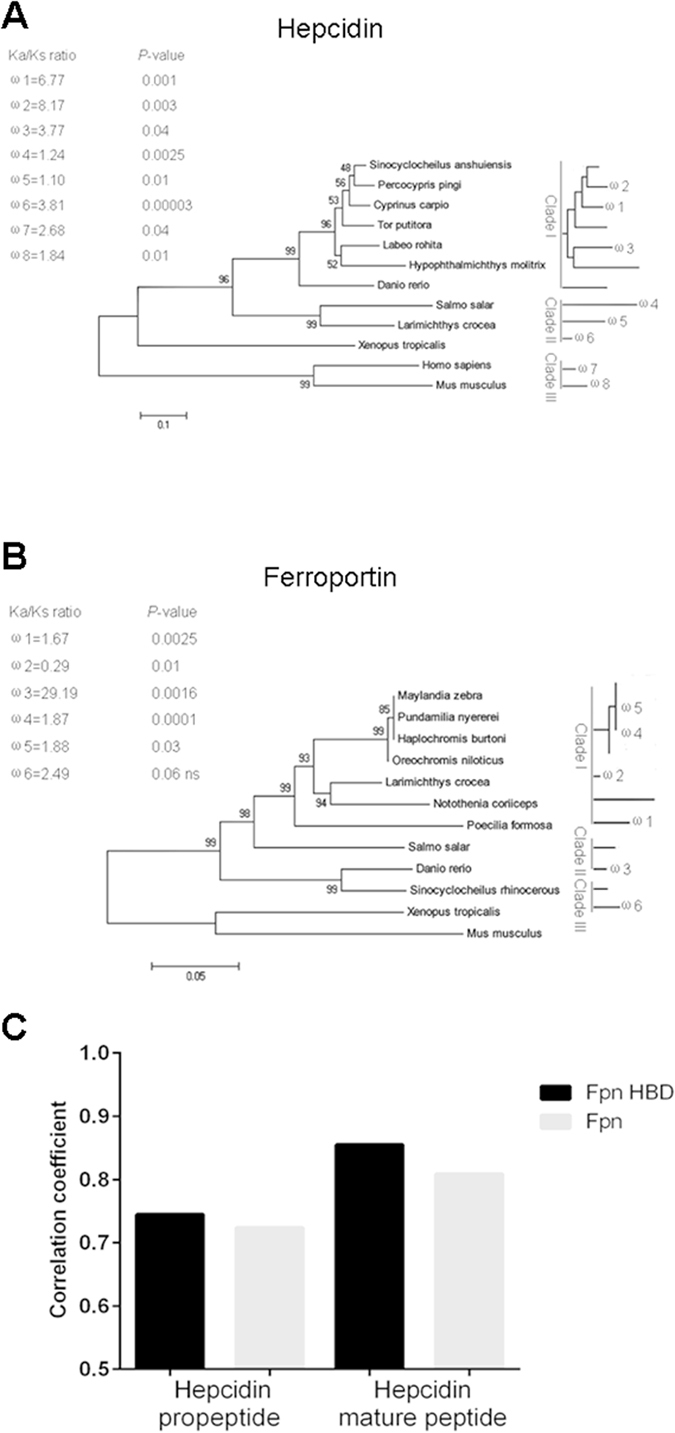
Co-evolution analysis of hepcidin and Fpn genes in vertebrates. (**A** and **B**) The 50% majority-rule consensus tree from Bayesian inference (BI) of conserved functional region of hepcidin (**A**) and Fpn (**B**) homologs among vertebrates were built, and each was divided into 3 clades based on the homologous relationship. The number above the branches indicates the statistical support value, while the scale bar indicates the number of substitutions per site and lineage-specific ω = Ka/Ks ratios for particular branches calculated by PAML. (**C**) The correlation coefficient of the relationship between hepcidin propeptide (or mature peptide) and Fpn (or Fpn-HBD).

The major mechanism of hepcidin in iron regulation is its inhibition of the cellular efflux of iron by the blockade of Fpn, which is a transmembrane iron channel that enables iron to shift from the cells into the circulation^7, 26^. Hepcidin has been found to directly interact with Fpn by binding to the hepcidin-binding domain (HBD) and inducing its internalization^27^. Considering the existence of the functional cooperation between hepcidin and Fpn, we speculated that a co-evolutionary relationship likely occurred between these molecules. In the 12 hepcidin and Fpn homologs that have highly conserved functional domains among vertebrates in this study^28^, the branch-specific Ka/Ks ratio of hepcidin ranged from 1.10 to 8.17 with an average of 3.67, and the Ka/Ks ratio of Fpn varied from 0.29 to 29.19 with an average of 5.82. Most of these values were >1 (Fig. 7A and B). Thus, positive selection was the predominant driving force of the hepcidin and Fpn evolution. Furthermore, the average correlation coefficient between Fpn and the hepcidin-binding domain (Fpn-HBD) was less than 0.75 or greater than 0.85. This finding suggested that hepcidin likely co-evolved with Fpn (predominantly with Fpn-HBD) under positive selection (Fig. 7C). Hepcidins were diverse in teleost fish, as determined by the observations that certain fish hepcidins serve as antimicrobial peptides, whereas other fish hepcidins function as both antimicrobial peptides and iron regulators^10, 29^. Thus, hepcidin initially participated as an antimicrobial peptide, and its hormone function in iron regulation may have originated from fish, which may be closely related to the diversity of aquatic environments with varying degrees of pathogen challenge, oxygenation, and iron concentration. This molecule acquired a dual function in the innate antibacterial immunity by possessing bactericidal and iron-mediated antimicrobial activities because of the adaptive evolution of hepcidin. The acquirement of the dichotomous functions of hepcidin may have been remarkably beneficial to the survival of vertebrate organisms during evolution and, thus, may have been retained for its conservation from fish to humans throughout vertebrate evolution.

CpG-ODNs with stimulatory CpG-motifs are effective stimulants of bacterial/viral genomic DNAs for the induction of immune responses through the TLR9 signaling pathway in both mammals and fish species^30, 31^, and they have been widely used as efficient immunological adjuvants in vaccinations^32^. In the present study, we showed that CpG-ODNs are also strong stimulants for hepcidin induction, which largely contributed to the host defence against bacterial infections. Thus, CpG-ODNs could be effective antibacterial agents for therapeutic applications against bacterial diseases. A precise feedback regulation between iron and hepcidin is present in humans and other mammals, as shown by the fact that the overloading of iron in the peripheral circulation results in a rapid induction of hepcidin in the liver, leading to the transportation of the extra iron into the storage cells^33^. Similarly, the present study has also clearly detected the induced upregulation of hepcidin in zebrafish by overloading iron into the circulation (Supplementary Fig. S1). This finding suggests that a conservative regulatory mechanism for iron and hepcidin existed during the vertebrate evolution. In addition, we provide further insights into the regulatory relationship between bacterial stimulation (by CpG-motif) and hepcidin expression through the CpG-TLR-9 signaling pathway. This study uncovers another important regulatory pathway that underlies pathogen recognition and hepcidin expression in the innate immunity, which provides a new connection between pathogen invasion, host defence, and iron metabolism in the complex regulatory network of pathogen-host interactions and iron homeostasis. A pathogen infection is closely related to hepcidin expression and iron homeostasis-relevant disorders^34, 35^. For example, an iron deficiency and iron excess are associated with cellular dysfunction, leading to various chronic diseases, such as hypoferremia, anemia of inflammation, and hereditary hemochromatosis^34, 36, 37^. Iron regulates the secretion of hepcidin, which in turn controls the iron concentration by decreasing Fpn on the cell surface^7^. Our study showed that hepcidin was also upregulated by the bacterial stimulation through a pattern recognition receptor (such as TLR-9)-mediated signalling pathway in zebrafish. This result suggests that a sustained infection may induce the overproduction of hepcidin, which can lead to hypoferremia and anemia of inflammation. From a host-defence perspective, this mechanism may be an adaptive response for an organism under a threat of pathogenic infection, favouring the host innate immunity against the bacterial invasion. Based on this notion, inflammatory signals or infectious stimulants, such as CpG-ODNs, may become strong boosters for the hepcidin-iron-mediated antimicrobial defence network. Otherwise, the clearance of inflammatory signals or the blockade of their signalling pathways, such as the TLR-9 signalling pathway in this case, may be of great clinical significance for the prevention of hypoferremia and anemia of inflammation.

Zebrafish are becoming an attractive model for exploring major issues in immunology and the disease mechanisms that are difficult to directly address in humans, for it possesses conserved innate immunity and numerous advantages for investigations. Emerging progress has been made in studies of developmental and comparative immunology and in modelling human diseases, including acute lymphoblastic leukemia, diabetes mellitus, alcoholic liver disorder, and hepatocellular carcinoma in this fish model^38–41^. In the present study, the zebrafish model showed its advantages for uncovering the dual function of hepcidin in the antimicrobial innate immunity by serving as an antibacterial peptide and an iron-regulatory hormone. To the best of our knowledge, this study is the first to provide experimental evidence revealing the antibacterial peptide and iron regulatory activities of hepcidin and its coordination in the innate immune defence against bacterial infections. Therefore, the zebrafish model, as a complement to mammalian models, will help to enhance our understanding of the molecular mechanisms of the hepcidin-mediated biological activities and diseases.

## Materials and Methods

### Zebrafish and bacterial strains

Zebrafish (*Danio rerio*) AB strain, weighing approximately 1.5–2 g with a body length of 2.5–3.5 cm, were kept in recirculating water at 28 °C under standard laboratory conditions^42^. *A. hydrophila* (BSK-10) and *V. alginolyticus* (ATCC 17749), which are two virulent pathogenic strains of infectious sepsis in a number of fish, were provided by the Zhejiang Institute of Freshwater Fisheries as previously described^42, 43^. The Top 10 strains of *E.coli* were preserved in our laboratory. The *A. hydrophila*, *V. alginolyticus* and *E.coli* strains were cultured in lysogeny broth (LB), LB agar, or M9 medium (M9 minimal salts medium containing 1% glucose and 0.3% Casein Tryptone) at 28 °C (for *A*. *hydrophila* and *V*. *alginolyticus*) or at 37 °C (for *E*. *coli*) as previously decribed^44^.

All animal care and experimental procedures were approved by the Committee on Animal Care and Use and the Committee on the Ethic of Animal Experiments of Zhejiang University. And we confirm that all methods were performed in accordance with the relevant guidelines and regulations.

### CpG-ODNs and bacterial genomic DNA

In total 19 CpG-ODNs (sequences shown in supplementary Table S1) with the classes A, B, and C stimulating motifs (GACGTT, GTCGTT, and AACGTT or GTCGTC) were synthesized with a phosphorothioated modification by Generay Biotechnology Company (Shanghai, China) followed by the method as described previously^45, 46^. These CpG-ODNs were dissolved in TE buffer (10 mM Tris-HCl, 1 mM EDTA, pH 8.0) at a concentration of 300 μg/mL. The *A. hydrophila* genomic DNA (*A. hydrophila* DNA) was extracted using the E.Z.N.A. Bacterial DNA Kit (LPS free, Omega Biotech) according to the manufacturer’s instructions. For this, the *A*. *hydrophila* cells at logarithmic growth phase were collected by centrifugation (8,000 g) at 4 °C for 10 min. After the cells were lysed by lysozyme and Proteinase K, the supernatant was carefully transferred to the HiBand DNA Mini column, followed by being washed twice with 70% ethyl alcohol. The *A*. *hydrophila* DNA was finally eluted by TE buffer (10 mM Tris-HCl, 1 mM EDTA, pH 8.0). Both of the CpG-ODNs and *A. hydrophila* DNA samples were stored at −30 °C, and diluted in phosphate buffer saline (PBS, pH 7.0) at specified concentrations before use.

### Serum samples with various treatments

The serum samples from the zebrafish that received different concentrations of the *A*. *hydrophila* DNA (0.5 μg, 1 μg, 3 μg, and 5 μg, dissolved in 10 μL of PBS, respectively) or CpG-ODNs (1.5 μg, dissolved in 10 μL of PBS) through intraperitoneal (i.p.) injections were prepared. The fish in the control groups received only mock PBS. The blood was collected 24 h after the stimulation, stored at room temperature for 1 h and at 4 °C for 4 h, and then centrifuged (10,000 g) at 4 °C for 10 min. The serum supernatants were collected and stored at −80 °C. In specified experiments, the serum samples that were derived from the *A*. *hydrophila* DNA or CpG-ODNs stimulated fish (named as stimulated serum) were subjected to various treatments as follows: 30 μL of the stimulated serum were incubated with anti-hepcidin Ab (diluted in PBS at 1:200 or 1:1,000) for 1 h at 37 °C; 30 μL of the stimulated serum were heat-treated to disrupt the activity of the complement components at 56 °C for 30 min; 30 μL of the stimulated serum with the neutralized and heat-incubated anti-hepcidin Ab were centrifuged (10,000 g) at 4 °C for 30 min and ultrafiltrated by a polyethersulfone membrane (3,000 MWCO, Sartorius) to remove the potential activities of hepcidin and the complement components; and the serum sample with the deprivation/inactivation of hepcidin and the complement components as described above was supplemented with 10 μM Fe^2+^ (supplement of 86.1 μM ferrous gluconate dihydrate that containing 10 μM Fe^2+^) to restore the iron concentration. These serum samples with various treatments were used in the following experiments.

### Preparation of Abs against hepcidin and OmpA

The mature peptide of the zebrafish hepcidin, which has 25 amino acids, and the antigenic epitope peptide of the *A. hydrophila* OmpA, which has 20 amino acids (ATLLSLVTPTVSVPTPTTRS) were chemically synthesized and purified through HPLC (C18 RP-HPLC) by the Bank Peptide Company (Hefei, China). The antigenic epitope peptide of OmpA was predicted by the online ABCpred and MHCPred programs^47^. Both of the hepcidin and OmpA peptides were coupled to keyhole limpet hemocyanin (KLH, Bank Peptide Company, Hefei, China) at a ratio of 10 mg:10 mg (carrier/peptide), respectively. Six weeks old male New Zealand white rabbits (~1.5 kg) were immunized with the KLH-coupled peptides (1 mg) in Complete Freund’s Adjuvant (CFA) on days 1 and 3 and in Incomplete Freund’s Adjuvant (IFA) on days 7 and 14, followed by the collection of anti-serum on day 28. The anti-hepcidin and anti-OmpA Abs were affinity isolated into IgG isotype by using a protein A agarose column (Qiagen, Suzhou, China) as previously described^42^. Western blot was conducted for the detection of Ab specificity as described in the Supplementary Information (Supplementary Fig. S2). The Ab titers were detected to be higher than 1:80,000 by ELISA using synthesized hepcidin and OmpA peptides adsorbed to the solid phase. The dilutions of Abs were 1:1,000 in all experiments unless otherwise indicated. The anti-GAPDH (glyceraldehyde-3-phosphate dehydrogenase) polyclonal Ab was purchased from Abcam (EPR16891, Abcam).

### Q-PCR for gene expression analysis

The transcripts of the hepcidin and OmpA genes were detected by Q-PCR. Total mRNA was extracted from the liver tissues of *A. hydrophila* DNA-stimulated or unstimulated fish using the TRizol reagent (Invitrogen). The total RNA derived from *A. hydrophila* strain that was cultured in the presence of the serum from the stimulated or unstimulated fish was extracted by the Spin Column Bacterial total RNA Purification Kit (Sangon Biotech). And the total RNA derived from *A. hydrophila* and zebrafish liver tissue were reverse-transcribed into cDNA using an RNA PCR Kit (AMV version 3.0, TaKaRa), Oligo-dT and random primers were used in reverse transcription followed by Q-PCR assays. All processes were performed according to the manufacturers’ instructions. Q-PCR was performed on a Mastercycler ep realplex machine (Eppendorf) using an SYBR Premix Ex Taq kit (TaKaRa). The Q-PCR was performed in a total volume of 10 µL. PCR program was 94 °C for 2 min, followed by 40 cycles at 94 °C for 20 s, 55 °C for 20 s, and 68 °C for 20 s. The relative hepcidin and OmpA steady state mRNA levels were calculated by the 2^−ΔΔCT^ method normalized to β-actin and 16s rRNA, respectively. The primers are shown in Supplementary Table S2. In all the cases, each sample was run in triplicate parallel reactions. The experiments were repeated independently at least three times.

### ELISA for the protein amount analysis

The amount of the hepcidin or OmpA protein in the serum or in *A. hydrophila* strain was examined by ELISA. The hepcidin protein was derived from the serum treated with various stimulations as described above. OmpA was prepared from the *A. hydrophila* strain with different treatments (incubated with the serum from the unstimulated fish, 1 μg *A. hydrophila* DNA stimulated fish, stimulated and 10 μM Fe^2+^ supplemented ones) with a lysis buffer (0.1% SDS, 1% Triton X-114, 1% sodium deoxycholate, 150 mM NaCl, 1 mM EDTA, 50 mM Tris-HCl, pH 7.4), containing a protease inhibitor mixture (Roche, USA)^48^. Each sample was coated onto microtiter wells overnight at 4 °C with the same protein concentration, which was determined by the Bradford method^49^. After blocking with 2% BSA for 2 h, the wells were incubated with the rabbit anti-hepcidin Ab or rabbit anti-OmpA Ab for 2 h at 37 °C, followed by incubation with the HRP-conjugated goat anti-rabbit Ab (Abcam, China) for 1 h at 37 °C. Then, the visualization was developed by a tetramethylbenzidine (TMB) substrate, and the absorbance (A_450_) value was read by Synergy H1 hybrid reader (BioTek, USA).

### Western blot analysis

Hepcidin, OmpA as well as the reference protein (GAPDH) sample was separated by 12% SDS-PAGE and transferred onto 0.45-μm polyvinylidene difluoride membranes (Bio-Rad Laboratories) for 90 min at 350 mA, and then blocked with TBST buffer (50 mM Tris-HCl, 150 mM NaCl and 0.1% Tween-20, pH 7.4) containing 5% non-fat dry milk (w/v) at 4 °C overnight. After washing with TBST three times for 30 min, the blots were incubated with rabbit anti-hepcidin Ab (1:1,000), rabbit anti-OmpA Ab (1:1,000) or rabbit anti-GAPDH Ab (Abcam. 1:3,000). The blots were washed thrice with TBST for 30 min, and then incubated for 1 h with the HRP-conjugated goat anti-rabbit IgG Ab (Abcam) at room temperature. After washing with TBST, the immunoreactive proteins were visualized using a chemical luminescent immunodetection system (Tanon 4500, Tanon Technology Company).

### Detection of serum-iron by ICP-MS

Each 100-μL serum sample with various treatments was applied to the inductively coupled plasma-mass spectrometry (ICP-MS, ELAN DRC-e, PerkinElmer) to detect the iron concentration^50^. The counts of ^56^Fe were collected successively using the medium resolution mode (*m*/Ä*m* = 3,000) for 0.8 s per data point. The operating parameters were set as follows: vacuum pressure was 5e–007 Torr, nebulizer gas flow (NEB) was 0.87 L/min, ICP RF power was 1,300 Watts, lens voltage was 7.5 Volts, analog stage voltage was 1,900 Volts, and pulse stage voltage was 1,000 Volts.

### *In vitro* bactericidal assay

The bactericidal activity of the serum treated with the *A*. *hydrophila* DNA (1 μg) stimulation and neutralization (incubated with anti-hepcidin Ab at 1:200 and 1:1,000) was examined by an *in vitro* inhibitory zone assay. *E. coli* and *A. hydrophila* strains were used in the experiment. The diameters of each inhibitory zone on the plates with an LB agar medium in plates (12 cm in diameter with 0.90 cm thickness) were measured by the agar diffusion method^51^. Each plate was smeared with a 50 μL solution of *E. coli* (3.0 × 10^6^ CFU) and *A. hydrophila* (2.0 × 10^5^ CFU) on the medium, and dotted with 30 μL of the serum samples with different treatments. The plates inoculated with *E. coli* and *A. hydrophila* were incubated at 37 °C and 30 °C, respectively, and the diameters of the inhibitory zone were detected 12 h after the incubation.

### Growth inhibition assay

The growth inhibition of *A*. *hydrophila* in the serum with various treatments was detected by CFU in an M9 medium. Five independent groups of *A. hydrophila* (2 mL) were cultured in the M9 medium at 30 °C with 30 μL of different serum samples (unstimulated, 1 μg *A. hydrophila* DNA stimulated, heated, anti-hepcidin Ab neutralized, and Ab neutralized plus 10 μM Fe^2+^ supplemented ones). *A. hydrophila* cultured alone in the M9 medium served as the control group. The Log_10_ CFU of *A. hydrophila* was detected at 0 h, 4 h, 8 h, 12 h, and 14 h after the incubation. The generation time (T_d_) was calculated during the exponential phase of growth according to the following formula:

$${{\rm{T}}}_{{\rm{d}}}=({{\rm{t}}}_{2}-{t}_{1})\times \{\mathrm{log}(2)/[\mathrm{log}({{\rm{q}}}_{2}{/q}_{1})]\},$$

where t_1_ and t_2_ represented the times and q_1_ and q_2_ represented the number of cells at t_1_ and t_2_, respectively^52^.

### *In vitro* complement attack assay

The tested *A. hydrophila* strain was washed twice with PBS to remove the trace amounts of nutrients, and resuspended in fresh M9 medium. Three independent serum samples (unstimulated, 1 μg *A. hydrophila* DNA stimulated, and stimulated plus 10 μM Fe^2+^ supplemented ones) were co-incubated with the test *A. hydrophila* strain. The complement attack assay was performed in polypropylene tubes containing 5% guinea pig serum (Mengry Bio-tech Co.) in a total volume of 2 mL in the M9 medium^53^. The CFU of each *A. hydrophila group* was calculated 12 h after the incubation.

### *In vivo* immunoprotection assay

The hepcidin-mediated antimicrobial activity was further evaluated by an *in vivo* immunoprotection assay. For this, the zebrafish were stimulated with the *A. hydrophila* DNA (1.5 μg per fish) for 24 h, followed by intraperitoneal (i.p) challenge with the A. *hydrophila* (2.1 × 10^5^ CFU per fish) or *V. alginolyticus* (2.9 × 10^5^ CFU per fish). In parallel, two control groups were included in this experiment. One control group received only mock PBS, which was not stimulated with *A*. *hydrophila* DNA but was challenged by the *A*. *hydrophila* or *V*. *alginolyticus* strains. Another control group received *A*. *hydrophila* DNA stimulation, but was not challenged by the two bacteria strains. Mortality in each group was monitored during the 5 day-period at 24 h intervals. The immunoprotection ratio (IPR) was calculated by the following formula: IPR = (1 – mortality of experimental group/mortality of control group) × 100%. The lethal dose 50 (LD_50_) was determined by challenging the stimulated fish with different dosages (1.0 × 10^5^, 4.1 × 10^5^, 8.4 × 10^5^, 1.2 × 10^6^, 1.6 × 10^6^, and 2.0 × 10^6^ CFUs per fish) of *V. alginolyticus* 24 h post-infection followed by the Bliss method^54^. The neutralization assay was performed to remove the protective hepcidin protein from the serum. For this, the zebrafish that received *A*. *hydrophila* DNA (1.5 μg per fish) stimulation were administrated with 10 μL anti-hepcidin Ab (1:100 diluted in PBS) followed by infections with *A*. *hydrophila* and *V*. *alginolyticus* as described above. In this assay, a negative control group was devised by administrating the fish with 10 μL non-specific rabbit IgG Ab (normal rabbit IgG polyclonal Ab from naive rabbit serum; 1:100 diluted in PBS; Merck Millipore, USA) instead of anti-hepcidin IgG Ab^42^. Each group in this study contained at least 30 fish, and each experiment was repeated independently at least 3 times.

### CpG-ODN-mediated protection assay

All 19 CpG-ODN candidates were subjected to an evaluation to determine whether a CpG-ODN can be a substitute for the bacterial DNA in eliciting the hepcidin-mediated antimicrobial immunity. The two known stimulatory CpG-ODNs (CpG-1670 and CpG-2006) and an inhibitory one (CpG-R) were used as the controls. ODN-C4, which has the strongest inducing effect on the expression of hepcidin, was independently administered to the fish in various concentrations (0.5 μg, 1 μg, and 1.5 μg), and its stimulatory effect on the hepcidin expression and hepcidin-mediated immune reactions were determined by Q-PCR, ELISA, Western blotting, increased serum bactericidal activity and decreased serum iron, which lead to an increase in the inhibition of bacterial growth and the sensitivity to a complement attack and enhanced the *in vivo* immunoprotective activity as described above.

### Co-evolutionary analysis of hepcidin and ferroportin

In total of 12 homologs sequences of hepcidin and Fpn were selected from NCBI using the BlastN online tool, and all sequences were aligned and edited using the Clustal X and MEGA 6.0 programs (GenBank accession numbers are listed in Supplementary Table S3). The phylogenetic relationship of these sequences among vertebrates was assessed using the Bayesian inference (BI) by the MRBAYES 3.1.2 program^55^. Maximum likelihood (ML) tree was constructed, and the node support was assessed using 1000 fast bootstrap replicates. The codeML package implemented in PAML 4.6^56^ and the KaKs Online Calculation Tool (http://kakscalculator.fumba.me/)^57^ were used to determine whether Ka/Ks (also known as ω or *d*N/*d*S) significantly differed in these two homologous sequences. A ratio of nonsynonymous (Ka) to synonymous (Ks) above 1 indicates that the positive selection is the predominant driving force of the gene evolution. Pearson’s correlation coefficient of the distance matrices between the ligand (hepcidin or its mature peptide) and receptor (Fpns or Fpn-HBDs) was also obtained^58, 59^. An *r* value above 0.8 was considered as the distinct correlation relationship.

### Statistical analysis

All experiments were repeated at least three times. The statistical evaluations of the differences between the means of the experimental groups were performed using Student’s *t* test, and the data were expressed as the mean ± SD. The criteria for statistical significance used were *p < 0.05, **p < 0.01, and ***p < 0.001 (GraphPad Prism 6.0). The overall survivals rates were presented as Kaplan-Meier estimates and analysed using the Breslow test. A P value of < 0.05 was considered indicative of statistical significance. The data were analysed with SPSS statistical software (version 19.0 for Windows, SPSS).

## Electronic supplementary material

Supplementary Information
