# Supplementary material for: Coordination of Bactericidal and Iron Regulatory Functions of Hepcidin in Innate Antimicrobial Immunity in a Zebrafish Model
Source: Sci Rep. 2017 Jun 27;7:4265. doi: 10.1038/s41598-017-04069-x (PMC5487360; doi:10.1038/s41598-017-04069-x)
Supplement: Supplementary file 1 — Supplementary Information [file 41598_2017_4069_MOESM1_ESM.pdf]

Coordination of Bactericidal and Iron Regulatory Functions of Hepcidin in Innate Antimicrobial Immunity in a Zebrafish Model

Xiao-feng Jiang[1], Zhi-fei Liu[1], Ai-fu Lin[1], Li-xin Xiang[1], and Jian-zhong Shao[1,2]

[1]College of Life Sciences, Zhejiang University, Hangzhou 310058, People's Republic of China; Key Laboratory for Cell and Gene Engineering of Zhejiang Province, Hangzhou 310058, People's Republic of China; [2]Laboratory for Marine Biology and Biotechnology, Qingdao National Laboratory for Marine Science and Technology, People's Republic of China

Supplementary Information

Induced-expression of hepcidin by overloading iron into zebrafish

It has been demonstrated that hepcidin can be induced in liver by *in vivo* overloading iron in humans and other mammalian models, which leading to the transportation of extra iron into storage cells. This is believed to be a feedback regulatory mechanism underlying maintenance of iron homeostasis. Similar to the performance of hepcidin in mammals, the zebrafish hepcidin can also be induced in liver by overloading extra iron (0.5 μM, 1 μM and 5 μM) into the fish, and the steady state level of hepcidin mRNA in liver and the amount of hepcidin protein in serum were upregulated in a dose-dependent manner (Fig S1). It suggests that the regulatory mechanism between hepcidin and iron was conserved from fish to mammals. Given another regulatory pathway exists between bacterial infection and hepcidin induction in zebrafish, it makes teleost fish an attractive model for the investigation of pathogen invasion, host defense and iron metabolism.

Detection of the specificity of anti-hepcidin Ab

The anti-hepcidin polyclonal Ab was produced in our labaratory. The protocol for preparation of the Ab was described in Materials and methods. The hepcidin protein samples were derived from serum, liver and kidney tissues; and the specificity of the anti-hepcidin Ab was examined by Western blot analysis. A clear single band with the expected molecular weight of hepcidin (approximately 9 kDa) was detected in every samples examined (Fig S2). The result indicated that the anti-hepcidin Ab was highly active and specific.

Correlation between OmpA expression and bacterial pathogenesis

To evaluate the correlation between OmpA expression and bacterial pathogenesis when iron levels in serum are manipulated, the *A. hydrophila* cells were cultured in the presence of normal serum derived from mock PBS-injected control fish, or in the presence of iron-deficiency serum derived from *A. hydrophila* DNA (1.5 μg) stimulated fish (for reducing OmpA protein expression on *A. hydrophila* cells, as shown in Fig 4). After a 12 h-incubation, the bacteria were centrifuged (10,000 g, 10 min at room temperature); then two groups of the bacteria were re-suspended and diluted to the same concentration by sterile PBS. Mortality was monitored during a 5 day-period after infection at 24 h intervals. Results showed that the zebrafish infected by *A. hydrophila* that was cultured in the presence of normal serum exhibited a severe disease progression with a significant lower survival, as determined by the dramatic decline in survival rate in each day post-infection (46.7% ±10.0%, 23.3% ± 3.3%, 16.7% ± 0%, 3.3% ± 3.3% and 0%). By contrast, zebrafish infected by *A. hydrophila* that was cultured in the presence of iron-deficiency serum exhibited a relatively smooth disease progression with a significant higher survival in each day post-infection (66.7% ±10.0%%, 50.0% ± 6.7%, 43.3% ± 6.7%, 30.0% ± 3.3% and

20.0% ± 3.3%). The results showed that the mortality of zebrafish infected by *A. hydrophila* with lower OmpA protein was significantly (P<0.05, Breslow test) declined compared with those fish infected with control *A. hydrophila* cells (Figure S 3).

Supplementary figures and tables

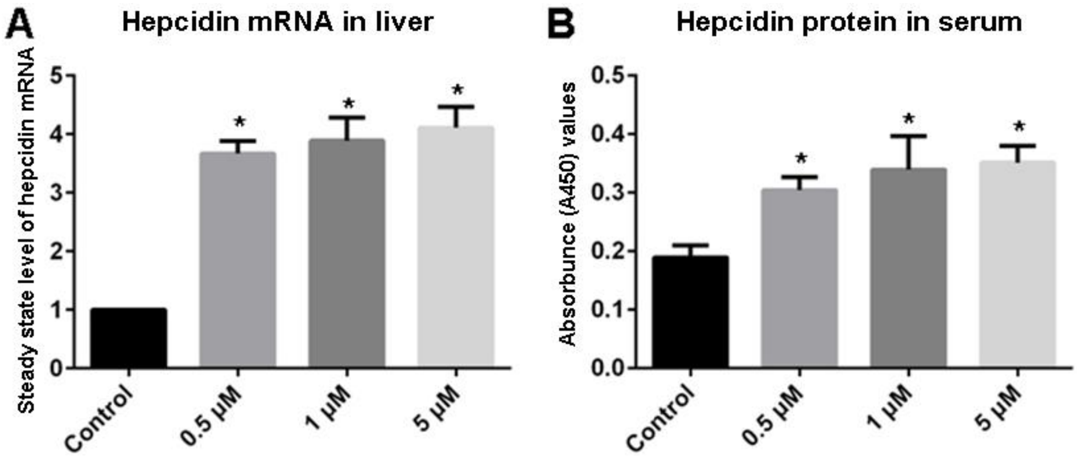

Figure S1. Detection of the steady state level of hepcidin mRNA in liver and the amount of hepcidin protein in serum in response to overloading of iron into zebrafish by Q-PCR (A) and ELISA (B). A significant difference was detected between each experimental group and control group, $^{*}p < 0.05$.

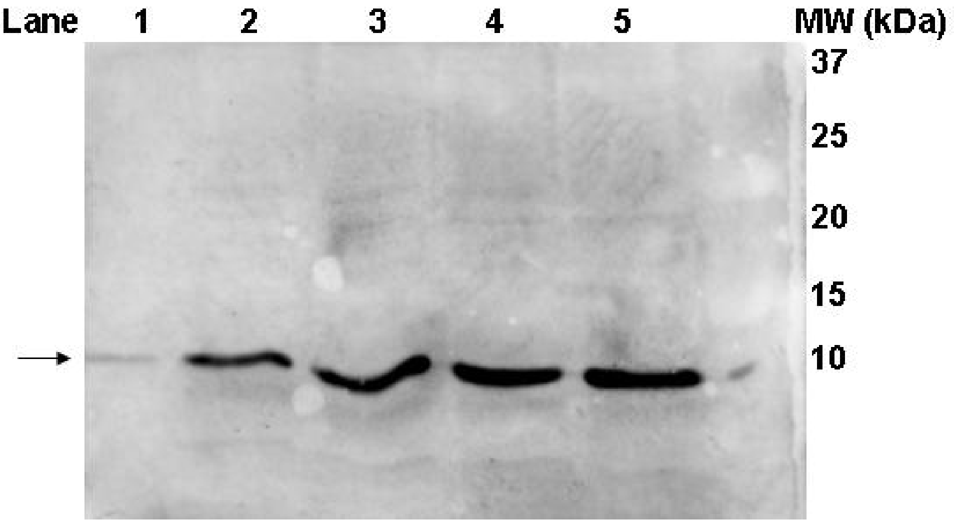

Figure S2. Detection of the specificity of anti-hepcidin Ab by Western blot analysis. Samples in lane1, lane 2 and 3, lane 4 and 5 were derived from kidney, serum and liver, respectively. The arrow pointed towards directly at the specific hepcidin band.

The figure showed here is full-length blots without any modification.

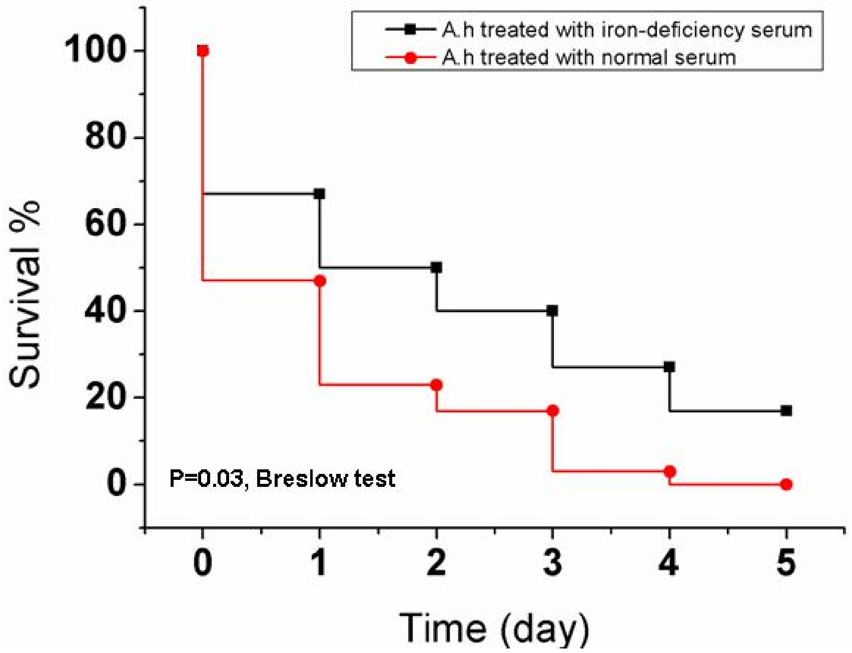

Figure S3. The survival rate of zebrafish infected by the *A*. *hydrophila* treated with iron-deficiency serum (black line) or normal serum (red line). The significant analysis performed by Breslow test was shown in the figure (P=0.03).

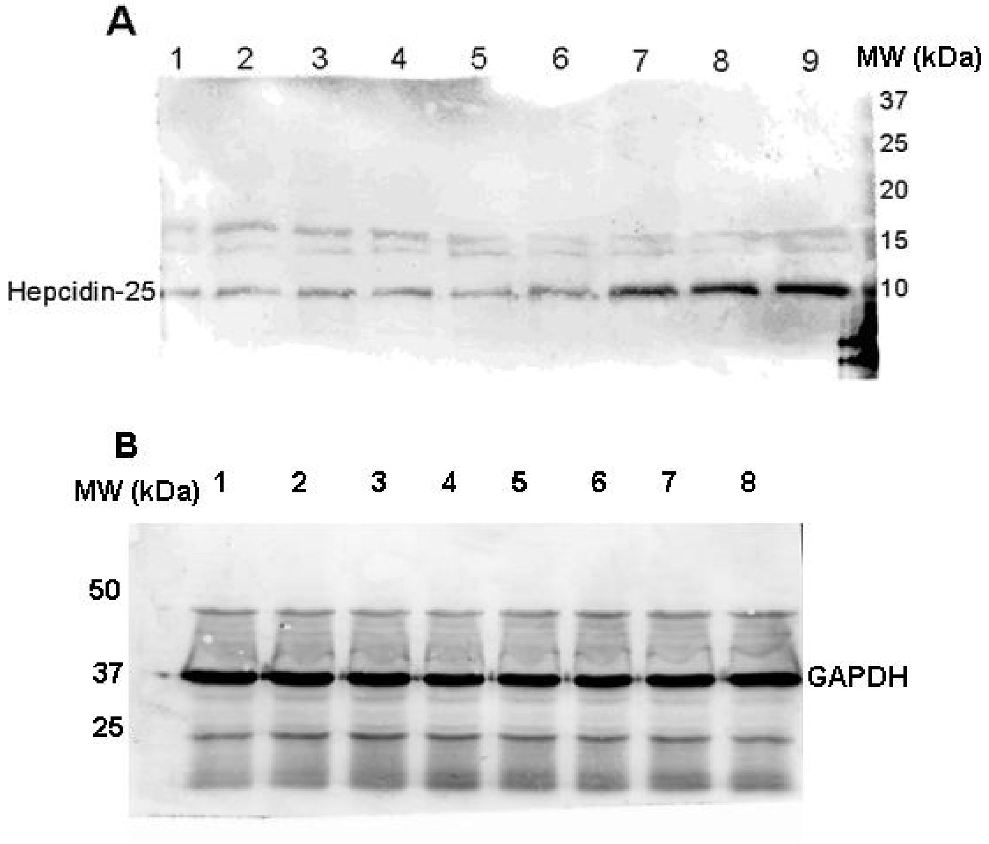

Figure S4. The full-length blots of Figure 1B. (A) Western blot analysis indicated the mature hepcidin (hepcidin-25) protein level in serum. Lane 1-5 were serum samples from unstimulated fish; Lane 6-9 were serum samples derived from *A.h* DNA stimulated fish (0.5 µg, 1 µg, 3 µg, 5 µg/fish); MW means molecular weights of the marker proteins. (B) Western blot analysis indicated the protein level of GAPDH. Lane 1-4 were serum samples from unstimulated fish; Lane 5-8 were serum samples derived from *A.h* DNA stimulated fish (0.5 µg, 1 µg, 3 µg, 5 µg/fish); MW means molecular weights of the marker proteins.

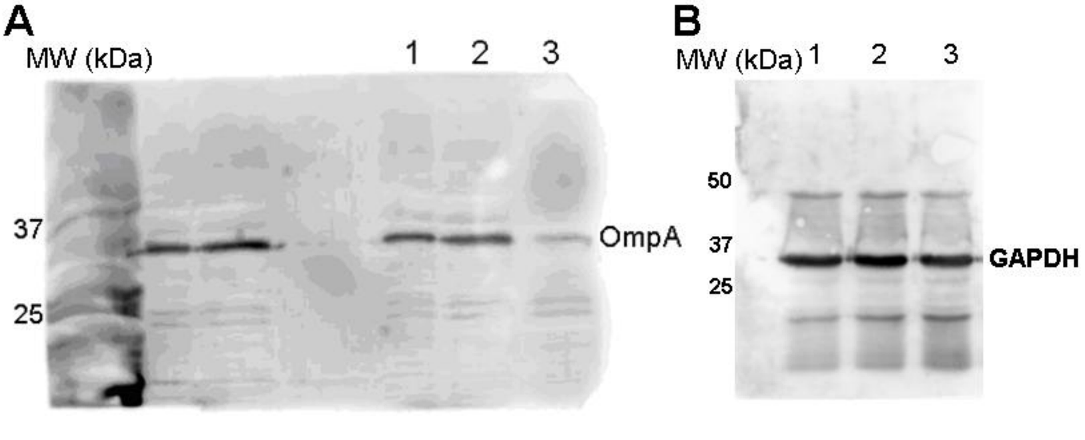

Figure S5. The full-length blots of Figure 4B and 4C. (A) Western blot analysis indicated the expression of OmpA protein. Lane 1 was protein sample from *A.*

hydrophila cells cultured with unstimulated serum; Lane 2 was protein sample from *A. hydrophila* DNA (1 μg) stimulated serum plus 10 μM Fe^{2+} supplemented ones; Lane 3 was protein sample from *A. hydrophila* cells cultured with stimulated serum; MW means molecular weights of the marker proteins. (B) Western blot analysis indicated the expression of GAPDH protein. Lane 1-3 were protein samples arranged in accordance with (A). MW means molecular weights of the marker proteins.

Table S1. Sequences of the synthetic CpG-ODNs

Name	CpG-ODN sequences
2006	**TCGTCGTTTTGTCGTTTTGTCGTT**
1670	ACCGATAACGTTGCCGGTGACG
R	ACCGATAAGCTTGCCGGTGACG
A1	**GG**TCGGACGTTCGA**GGGGGG**
A2	**GG**TCGGTCGTTCGA**GGGGGG**
A3	**GG**TCGAACGTTCGA**GGGGGG**
A4	**GG**TCGGTCGTCCGA**GGGGGG**
A5	**GG**TCGACGGGTCGA**GGGGGG**
B1	**TCGTCGGACGTTACGTCGTT**
B2	**TCGTCGGTCGTTACGTCGTT**
B3	**TCGTCGAACGTTACGTCGTT**
B4	**TCGTCGGTCGTCACGTCGTT**
B5	**TCGTCGACGGGTACGTCGTT**
C1	**TCGTCGTCGATCGATCGAGTCGTT**
C2	**TCGTCGTCGACCGGTGCAGTCGTT**
C3	**TCGTCGTCGATCGATGCAGTCGTT**
C4	**TCGTCGTCGAGCGAGGCAGTCGTT**
C5	**GTCGTTTCGACGGGTGCAGTCGTT**
C6	**TCGTCGGACGTTTCGAGTCGTT**
C7	**TCGTCGGTCGTTTCGAGTCGTT**

C8	**TCGTCGAACGTTTCGAGTCGTT**
C9	**TCGTCGGTCGTCTCGAGTCGTT**

Note: The phosphorothioate modified dinucleotides are indicated in bold letters.

Table S2. Primers sequences used in PCR

Primer Name	Sequences and lenth (5'-3')
Hepcidin sense	CCAGATCACAGCCGTTCCCTTC (22nt)
Hepcidin antisense	GAGATGCAGCCTGCATTTATACCC (24nt)
β-actin sense	ACACCTTCTACAATGAGCTG (20nt)
β-actin antisense	CTGCTTGCTGATCCACATCT (20nt)
OmpA sense	ACCGAATTCATGGCAACGCTGTTG (24nt)
OmpA antisense	GATCTCGAGTCACGCCGTCACACT (24nt)
16s rRNA sense	AGAGTTTGATCATGGCTCAG (20nt)
16s rRNA antisense	TACGGATACCTTGTTACGACTT (22nt)

Table S3. Information on the genes of hepcidin and Fpn among each individual species used for phylogenic analyses.

Gene Name	Species	Accession Numbers
Hepcidin	*Danio rerio*	NP_991146.1
	Cyprinus carpio	AFY23859.1
	Percocypris pingi	AIN25990.1
	Hypophthalmichthys molitrix	AGT19511.1
	Larimichthys crocea	ABC18307.1
	Salmo salar	XP_014025519.1
	Xenopus tropicalis	NP_001090729.1
	Homo sapiens	NP_066998.1
	Mus musculus	NP_115930.1
Ferroportin (Fpn)	*Xenopus tropicalis*	NP_001090746.1
	Mus musculus	NP_058613.2
	Danio rerio	AAI63874.1
	Sinocyclocheilus rhinocerous	XP_016394745.1
	Salmo salar	XP_014019727.1
	Larimichthys crocea	KKF14475.1
	Pundamilia nyererei	XP_006807681.1

Haplochromis burtoni	XP_018552216.1
Oreochromis niloticus	XP_003458401.1
Maylandia zebra	XP_004559291.1
Notothenia coriiceps	XP_010786941.1
Poecilia formosa	XP_007541658.1
